# Acute exercise mobilizes CD8^+^ cytotoxic T cells and NK cells in lymphoma patients

**DOI:** 10.3389/fphys.2022.1078512

**Published:** 2023-01-11

**Authors:** Tiia Koivula, Salla Lempiäinen, Petteri Rinne, Maija Hollmén, Carl Johan Sundberg, Helene Rundqvist, Heikki Minn, Ilkka Heinonen

**Affiliations:** ^1^ Turku PET Centre, University of Turku and Turku University Hospital, Turku, Finland; ^2^ Institute of Biomedicine, University of Turku, Turku, Finland; ^3^ MediCity Research Laboratory, University of Turku, Turku, Finland; ^4^ Department of Physiology and Pharmacology, Karolinska Institutet, Stockholm, Sweden; ^5^ Department of Learning, Informatics, Management and Ethics, Karolinska Institutet, Stockholm, Sweden; ^6^ Department of Laboratory Medicine, Karolinska Institutet, Stockholm, Sweden; ^7^ Department of Oncology and Radiotherapy, Turku University Hospital, Turku, Finland

**Keywords:** acute exercise, immune cells, mobilization, cancer, lymphoma, physical activity, white blood cells, immunology

## Abstract

**Background:** Studies have shown that acute exercise can mobilize several leukocyte subpopulations in healthy individuals. Our aim was to investigate whether a 10-min acute exercise has an effect on immune cell proportions in lymphoma patients.

**Methods:** This study included seven lymphoma patients referred to curative oncologic therapy. Three had Hodgkin and four non-Hodgkin lymphoma, one was female, and their mean age was 51. Patients underwent a 10-min acute exercise on a bicycle ergometer at moderate exercise intensity. Whole blood samples were taken at rest, immediately after exercise, and 30 min after exercise. Leukocyte subpopulation levels were determined using flow cytometry.

**Results:** Proportions of total NK cells and CD56^+^CD16^+^ NK cells of total leukocytes increased immediately after exercise and decreased back to baseline at 30 min post-exercise. Proportion of CD8^+^ T cells of total T cells increased and proportion of CD4^+^ T cells of total T cells decreased immediately after exercise, and both returned to baseline at 30 min post-exercise. There was no change in the proportions of B cells, granulocytes, or monocytes. Exercising diastolic blood pressure correlated positively with changes in total NK cell and CD56^+^CD16^+^ NK cell proportions, and exercising mean arterial pressure correlated positively with change in CD56^+^CD16^+^ NK cell proportion.

**Conclusion:** Our findings indicate that a single acute exercise bout of only 10 min can cause leukocytosis in lymphoma patients, particularly on cytotoxic T cells and NK cells, which are the most important immune cells fighting against cancer.

## Introduction

Lymphoma is a cancer of the lymphatic system that is traditionally divided into Hodgkin and non-Hodgkin lymphomas. In 2020 approximately 630,000 people were diagnosed with lymphomas worldwide and approximately 280,000 people died of lymphomas the same year (The Global Cancer Observatory, World Health Organization, 2020). The rapid development of oncologic treatments in recent decades has led to a reduction in mortality, but research needs to be continued because e.g., the most common subtype, diffuse large B-cell lymphoma, which represents 30%–40% of all cases, has a 5-year survival rate of 60%–70% (Li et al., 2018).

Regular physical exercise has been associated with reduced cancer mortality in many types of cancer, including lymphoma (Pophali et al., 2018). Exercise not only improves cancer patients’ ability to function, sleep quality, and body composition (Streckmann et al., 2014; Schmitz et al., 2010), but recent research suggests that it also has a direct effect on tumor biology. In preclinical studies, exercise stress has been shown to control cancer growth and metastasis as well as to improve the efficacy of cancer therapies (Pedersen et al., 2016; Hojman et al., 2018; Wennerberg et al., 2020). These positive effects of exercise are likely mediated by several different mechanisms, one of which is through immunomodulation (McTiernan, 2008; Rundqvist et al., 2020). Cytotoxic T cells and natural killer (NK) cells play an important role in the defense against tumor cells (Waldhauer and Steinle, 2008; Raskov et al., 2021). They both respond to acute exercise in healthy individuals as their numbers increase in the bloodstream (Edwards et al., 1984). The phenomenon is transient, as the number of these cells decreases to baseline levels or even lower quickly after acute exercise, when the cells are thought to migrate into tissues (Pedersen and Hoffman-Goetz, 2000; Krüger et al., 2008). In addition, the activity of cytotoxic T cells and NK cells seems to increase during exercise (Edwards et al., 1984; Nielsen et al., 1996), not only because of the immune cell recruitment, but also because exercise changes cytokine profile and causes favorable epigenetic modifications in immune cells (Zimmer et al., 2015). Deficiency of cytotoxic T cells and NK cells in lymphoma patients is associated with poorer outcomes (Xu-Monette et al., 2019), indicating the importance of cell count. The immune cell-enhancing effect of exercise may therefore be important for this patient group.

The effect of acute exercise on immune cells has been widely studied in healthy volunteers (Edwards et al., 1984; Tvede et al., 1989; Shinkai et al., 1992; Shek et al., 1995; Rowbottom and Green, 2000). In lymphoma patients, Zimmer et al., (Zimmer et al., 2014) as well as Verma et al., (Verma et al., 2009) have studied the effect of aerobic exercise on cytokine levels. However, to the best of our knowledge, there is no research data on immune cell mobilization in this patient group. It is important to study the biological mechanisms of acute exercise in cancer patients, as findings from such studies may be of importance when designing larger clinical trials. Understanding immune cell functions in response to acute exercise could also be used to modify more suitable exercise recommendations for cancer patients and to learn how to use exercise as part of cancer treatments.

Consequently, the aim of this study was to determine whether acute exercise mobilizes immune cells, and if so which subpopulations, in the circulation of lymphoma patients.

## Methods

This study was conducted at the Turku PET Centre, Turku, Finland between September 2020, and July 2021. Patients participated voluntarily by signing an informed consent form after reviewing the study information sheet and hearing an explanation about the study from the investigators. Good clinical practice and the Declaration of Helsinki were followed. The study was approved by the Ethics Committee of the Hospital District of Southwestern Finland and is registered in the international register of clinical trials (Clinicaltrials.gov NCT03987724).

### Participants

Recently diagnosed Hodgkin and non-Hodgkin lymphoma patients with a tumor in the neck or mediastinal area were selected for this study. Exclusion criteria were abnormal fatigue, anemia, or some physical dysfunction due to disease. A total of seven lymphoma patients (six males, one female) were recruited from Turku University Hospital. Mean age was 51 (SD 21) years and mean body mass index (BMI) was 26.9 (SD 3.9) kg/m^2^. Two of the lymphoma patients had diffuse large B-cell non-Hodgkin lymphoma. One patient had Ann Arbor stage IA and IPI score 2, while the other had Ann Arbor stage IVA and IPI score 4 diffuse large B-cell lymphoma. Both diffuse large B-cell lymphomas had been transformed, one from the follicular lymphoma and the other from nodular marginal zone lymphoma. Three of the lymphoma patients had Hodgkin`s disease. One of them were advanced favorable Hodgkin lymphoma nodular sclerosis with Ann Arbor score IVB and IPS risk score 3. One had advanced unfavorable Hodgkin lymphoma nodular sclerosis with Ann Arbor stage IIB and IPS risk score 4, and one had early unfavorable Hodgkin lymphoma nodular sclerosis, Ann Arbor stage IIA with IPS risk score 3. One of the lymphoma patients had follicular lymphoma grade 1–2, stage IIIA, FLIPI-score 3, and one had stage IIIA small lymphocytic lymphoma (Cheson et al., 2014). The study was performed before the start of patients’ cancer treatments and did not cause any delay in treatments.

### Study design

Patients visited the study laboratory at least 1 day, typically more than 24 h before the study day (ranging from 1 week to 18 h) during which they tested pedaling a supine bicycle ergometer. During that subjective testing a pedaling power for the actual study visit was determined according to each patient’s own perceived fitness. Thus, testing was started with minimal power production and was gradually increased until the patient was confident that he/she could cycle with the chosen power for 10 min without fatigue development. Higher power production levels were also tested to make sure that the choice was correct, and patient could not anymore cycle for several minutes with the higher intensity. During that test it was also determined that heart rate was at least modestly increased from rest, typically above 100 bpm. Strong physical exertion, alcohol consumption and caffeine were prohibited for at least 24 h prior to the study day. On the study day, patients conducted a 10-min bicycle exercise with a specific supine cycle ergometer (Tunturi E30^R^, Hungary). Prior to starting the measurements, an intravenous catheter was inserted for repeated blood sampling. From each patient, three 10 mL peripheral vein blood samples were taken to EDTA tubes (BD Biosciences, San Jose, United States) at rest, immediately after exercise (within 1-2 min) and 30 min after exercise. Heart rate (Palmsat 2500, Nonin, Plymouth, United States) and blood pressure (Apteq AE701f, Rossmax Swiss GmbH, Berneck, Switzerland) were measured at rest and during exercise. Rate pressure product (RPP) was measured multiplying systolic blood pressure by heart rate. Mean arterial pressure (MAP) was measured by doubling the diastolic blood pressure and adding the sum to the systolic blood pressure and dividing that sum by 3. Age predicted maximum heart rate was measured by subtracting age from 220. Patients’ sensing of strenuousness of the exercise was determined with Borg scale.

### Flow cytometry

For flow cytometry, patients’ blood samples were analyzed within 5 h of sampling. If the samples were not processed immediately, whole blood samples were stored in EDTA tubes in refrigerator between sampling and staining. Fc Block (BD Biosciences, San Jose, United States) was added to 100 µL of whole blood and incubated for 10 min at room temperature. Thereafter, the samples were stained with fluorophore-labeled CD monoclonal antibodies (BD Biosciences, San Jose, United States). The CD antibodies used were CD45-FITC (clone HI30), CD3-BV605 (clone UCHT1), CD4-BV421 (clone RPA-T4), CD8-BV786 (clone RPA-T8), CD16-PE (clone 3G8), CD19-PE-CY7 (clone HIB19), and CD56-APC (clone NCAM162) for panel one and CD45-FITC (clone HI30), CD3-BV605 (clone UCHT1), CD14-APC (clone M5E2), CD16-PE (clone 3G8), CD19-PE-CY7 (clone HIB19), and CD64-BV421 (clone 10.1) for panel two. Samples were incubated for 20 min in the dark at room temperature. Red blood cells were lysed with 1X FACS lysing solution (BD Biosciences, San Jose, United States) and incubated for 10 min in the dark at room temperature. Samples were washed with PBS (Thermo Fisher Scientific, Waltham, United States). After centrifugation, the cells were resuspended in 300 µL of PBS and samples were pipetted into a flat bottom 96-well plate. As applied previously (Rinne et al., 2018; Viitala et al., 2019; Kadiri et al., 2020; Kadiri et al., 2021; Tadayon et al., 2021), samples were analyzed by running 150 µL of each sample with a BD LSR Fortessa™ flow cytometer (BD Biosciences, San Jose, United States) on the same day using BD FACSDiVa v.8 program (BD Biosciences, San Jose, United States). All samples were analyzed by the same person under the same conditions.

### Statistical methods

Flow cytometry data was analysed with Flowjo (BD Biosciences, San Jose, United States). Repeated measurement ANOVA was used to test the changes in cell proportions. When the main effect (time) was less than *p* < .05, statistical differences in time points were considered by Tukey-post-hoc test. The associations between variables were examined by Pearson’s correlation. Significance was determined at *p* < .05. All statistical analyses were performed with Graphpad prism 8.0.

## Results

### Exercise characteristic

Exercising power production was on average 62 ± 22 W. Strenuousness of the exercise was on average 13 ± 2 on Borg scale. Heart rate was 77 ± 15 bpm at rest and increased significantly to 109 ± 21 bpm during exercise (*p* = 0,001). A heart rate percentage from age predicted maximal heart rate was 65 ± 13% during exercise. Systolic blood pressure was 129 ± 15 mmHg at rest and increased significantly to 155 ± 31 mmHg during exercise (*p* = 0,034) and diastolic blood pressure was 69 ± 9 mmHg at rest and 84 ± 23 mmHg during exercise. MAP was 89 ± 11 mmHg at rest and 128 ± 77 mmHg during exercise. RPP was 9,894 ± 2,065 bpm • mmHg at rest and increased significantly to 16,883 ± 4,391 bpm • mmHg during exercise (*p* = 0,007).

### Immune cell response to acute exercise

**FIGURE 1:**
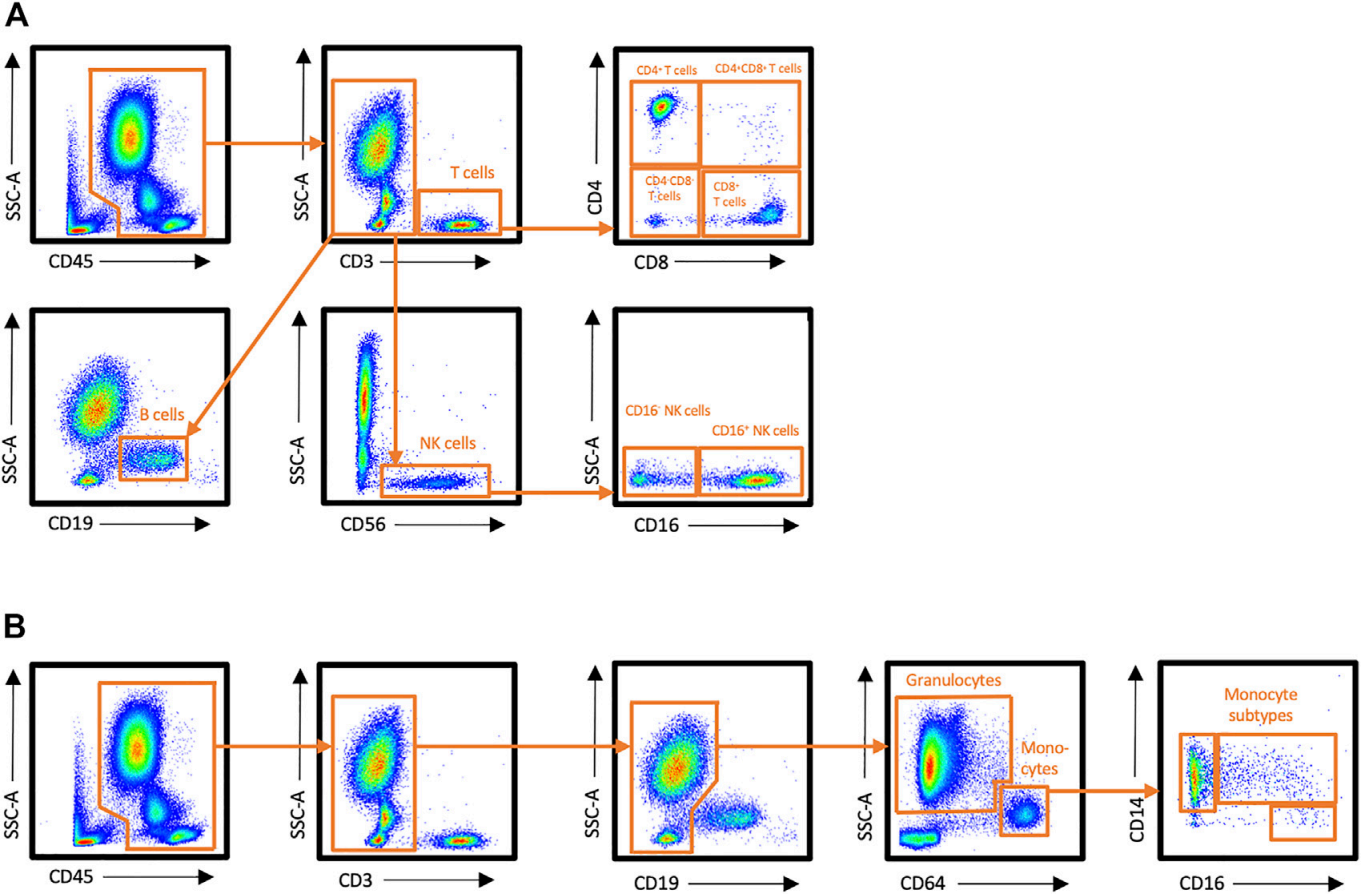
Flow cytometry gating strategy for leukocyte subsets in peripheral blood. **(A)** Staining panel 1, **(B)** Staining panel 2.

Number of total leukocytes, and proportions of T cells, B cells, NK cells, granulocytes, and monocytes were examined in peripheral blood at rest, immediately after exercise, and 30 min after exercise with flow cytometry. Gating strategy is presented in Figure 1.

**FIGURE 2:**
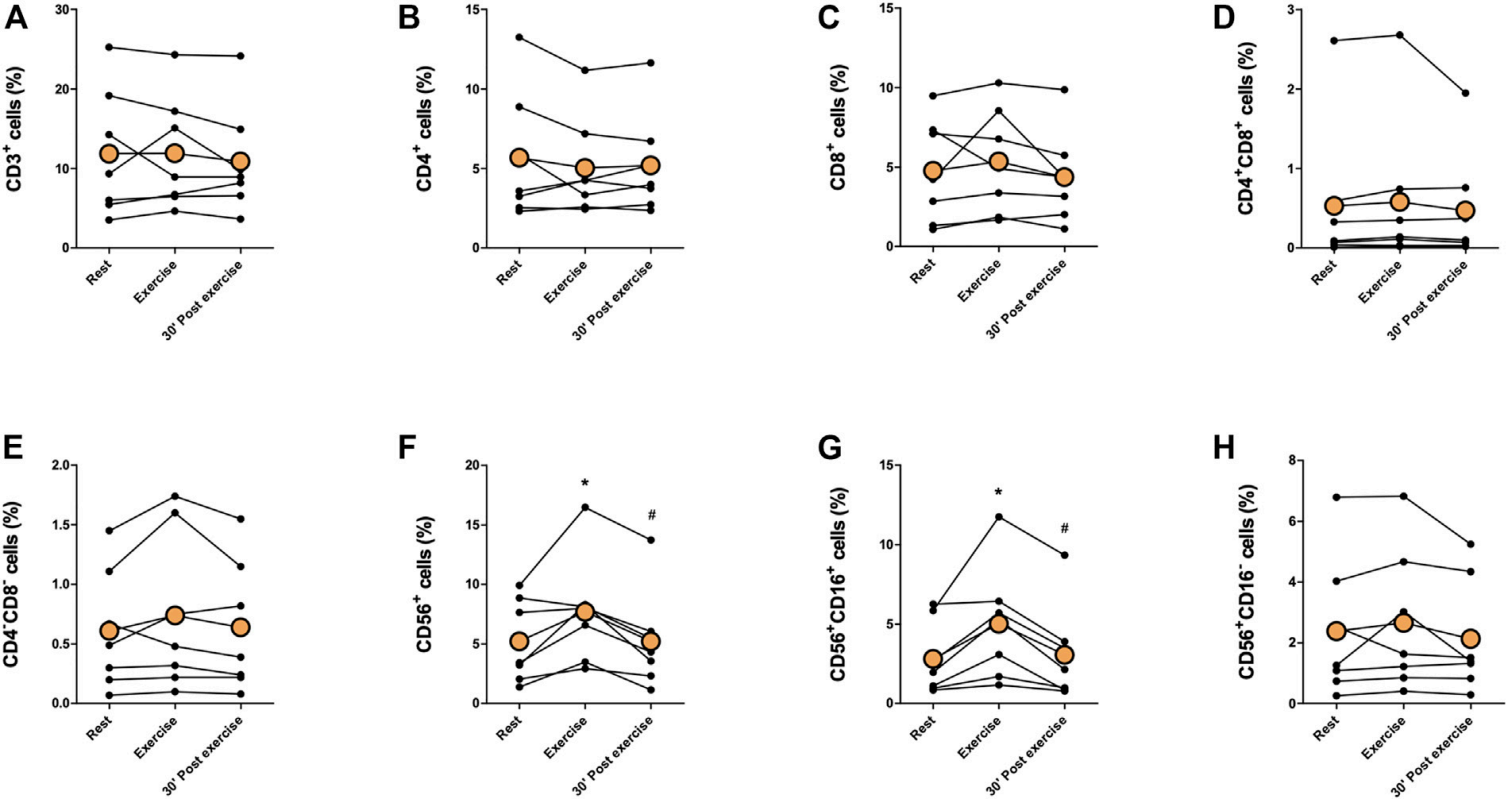
Changes in proportions of **(A)** total T cells, **(B)** CD4^+^ T cells, **(C)** CD8^+^ T cells, **(D)** CD4^+^CD8^+^ T cells, **(E)** CD4^−^CD8^−^ T cells, **(F)** total NK cells, **(G)** CD56^+^CD16^+^ NK cells, and **(H)** CD56^+^CD16^−^ NK cells of total leukocytes with acute exercise. Orange points represent the mean. *p < 0,05 between rest and exercise; #*p* < 0,05 between exercise and 30 min post-exercise.

**FIGURE 3:**
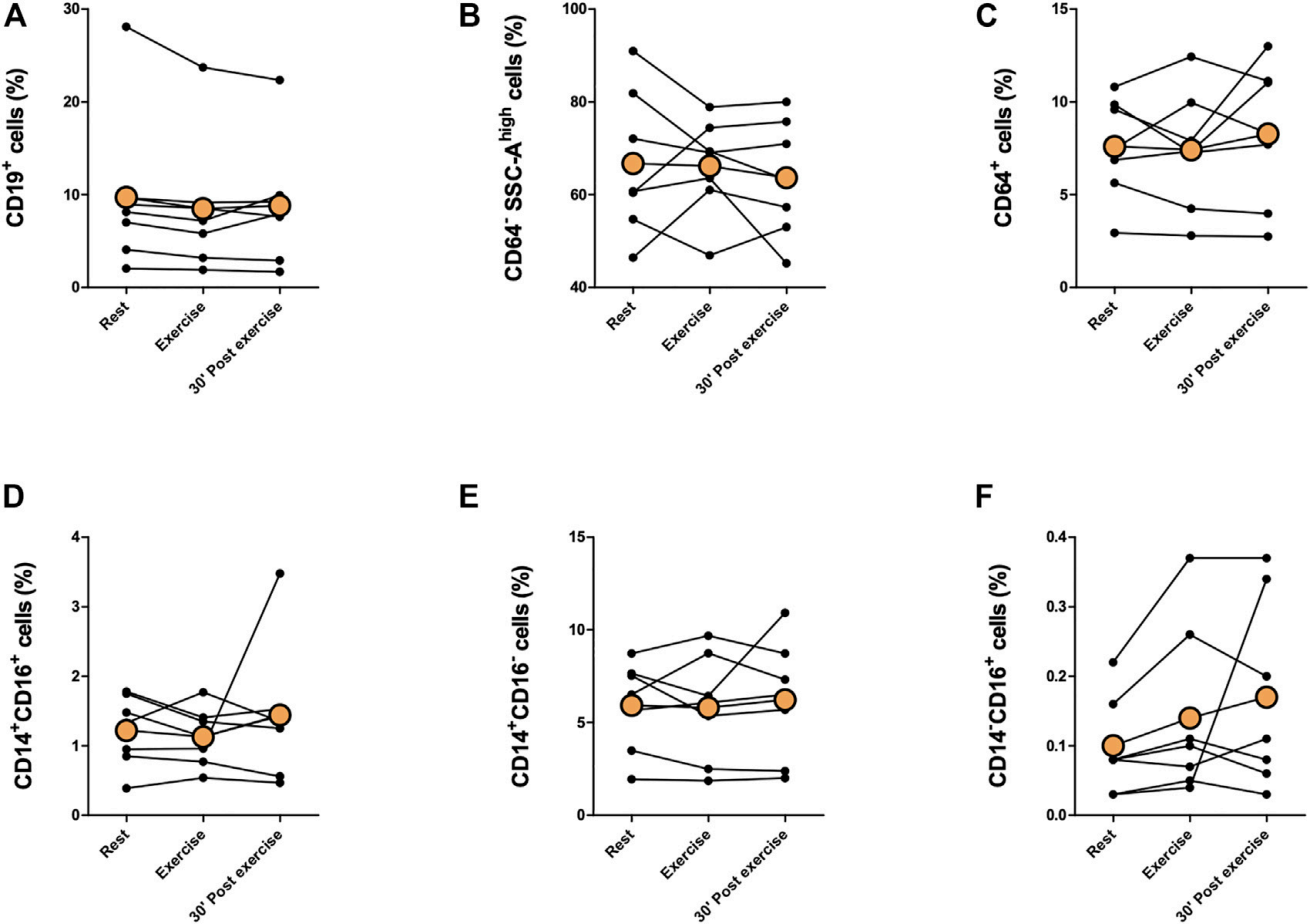
Changes in proportions of **(A)** CD19^+^ B cells, **(B)** granulocytes, **(C)** total monocytes, **(D)** CD14^+^CD16^+^ monocytes, **(E)** CD14^+^CD16^−^ monocytes, and **(F)** CD14^−^CD16^+^ monocytes of total leukocytes with acute exercise. Orange points represent the mean.

**FIGURE 4:**
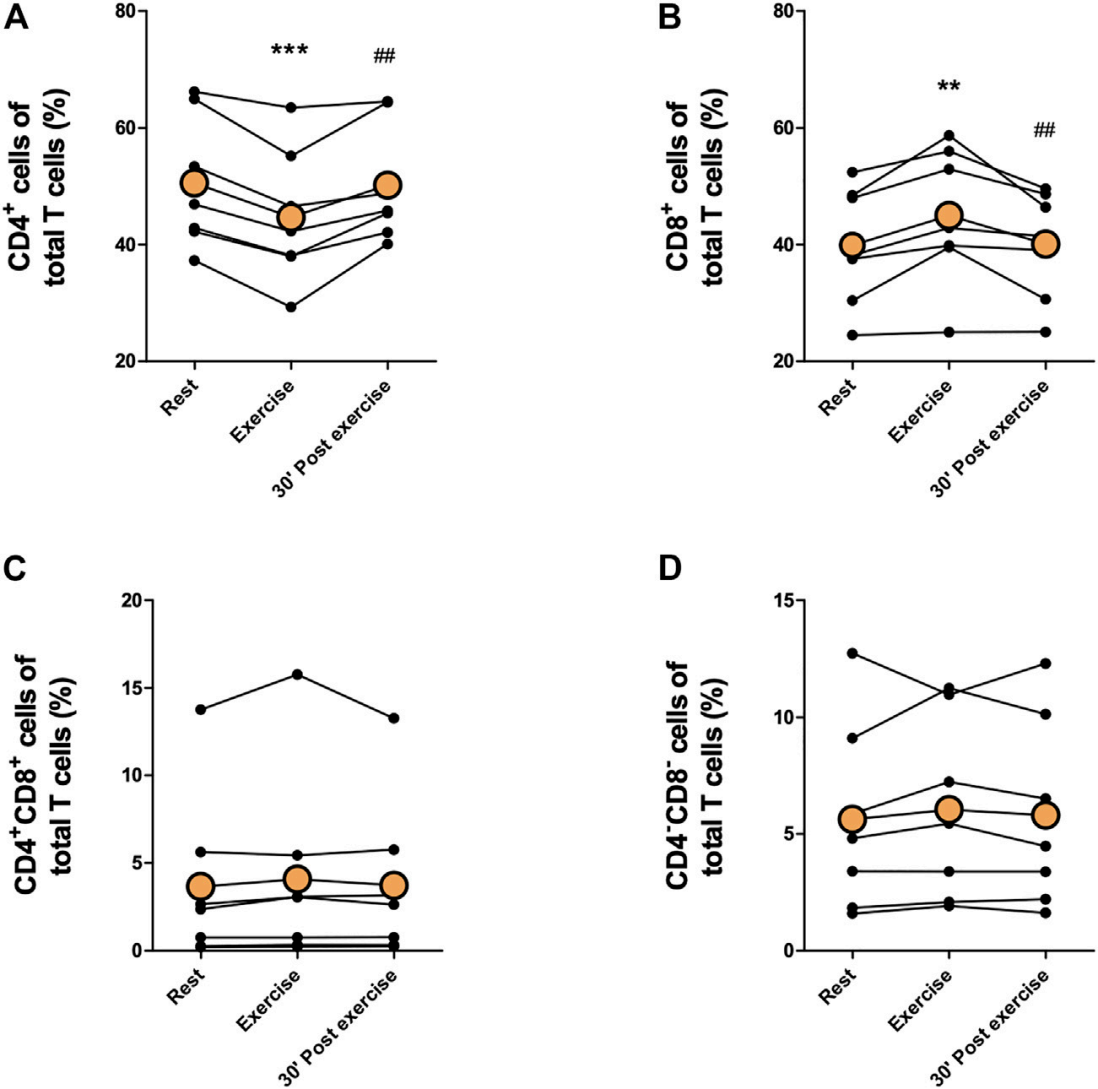
Changes in proportions of **(A)** CD4^+^ T cells, **(B)** CD8^+^ T cells, **(C)** CD4^+^CD8^+^ T cells, and **(D)** CD4^−^CD8^−^ T cells of total T cells with acute exercise. Orange points represent the mean. **p < 0,01 between rest and exercise; ***p < 0,001 between rest and exercise; ##*p* < 0,01 between exercise and 30 min post-exercise.

Proportions of immune cells of total leukocytes are presented in Figures 2, 3. Proportions of total T cells, CD4^+^ T cells, CD8^+^ T cells, CD4^+^CD8^+^ T cells, or CD4^−^CD8^−^ T cells of total leukocytes did not change in response to exercise (Figures 2A–E). The proportions of total NK cells and CD56^+^CD16^+^ NK cells of total leukocytes increased significantly immediately after exercise (*p* < 0,05) and decreased between exercise and 30 min post-exercise (*p* < 0,05), but the proportion of CD56^+^CD16^−^ NK cells did not change (Figures 2F–H). Further, there was no change in the proportions of CD19^+^ B cell, granulocytes, or monocytes of total leukocytes (Figure 3.) The proportion of CD4^+^ T cells of total T cells decreased immediately after exercise (*p* < 0,001) and increased between exercise and 30 min post-exercise (*p* < 0,01) while the proportion of CD8^+^ T cells of total T cells increased immediately after exercise (*p* < 0,01) and decreased between exercise and 30 min post-exercise (*p* < 0,01) (Figures 4A, B). The proportions of CD4^+^CD8^+^ T cells or CD4^−^CD8^−^ T cells of total T cells did not change (Figures 4C, D).

Changes in absolute immune cell counts with methodological considerations in response to exercise are presented in Supplementary Figures S1–S3.

### Correlation analyses

Correlations between age, BMI, and exercise intensity variables and change in cell proportions are presented in Table 1. Age and BMI correlated positively with change in granulocyte proportion. Exercising systolic blood pressure correlated positively with changes in granulocyte, total monocyte, and CD14^+^CD16^+^ monocyte proportions and negatively with changes in CD4^+^ T cell and CD19^+^ B cell proportions. Exercising diastolic blood pressure correlated positively with changes in CD4^+^CD8^+^ T cell, total NK cell, CD56^+^CD16^+^ NK cell, and CD14^−^CD16^+^ monocyte proportions and negatively with change in CD19^+^ B cell proportion. Further, heart rate percentage of age predicted maximal heart rate correlated positively with change in CD4^−^CD8^−^ T cell proportion. Exercising rate pressure product correlated positively with change in CD14^−^CD16^+^ monocyte proportion and negatively with change in CD19^+^ B cell proportion. Exercising mean arterial pressure correlated positively with changes in CD4^+^CD8^+^ T cell, CD56^+^CD16^+^ NK cell, CD14^+^CD16^+^ monocyte, and CD14^−^CD16^+^ monocyte proportions and negatively with change in CD19^+^ B cell proportion. Lastly, pedaling power (watts) correlated negatively with change in CD4^+^CD8^+^ T cell proportion. Same correlation analysis with change in absolute cell counts are presented in Supplementary Table S1. Moreover, correlation between total leukocyte counts determined with Sysmex XN analyser and with flow cytometry is presented in Supplementary Material.

**TABLE 1:** Pearson correlation between age, BMI, and exercise intensity variables and change in cell proportions between rest and exercise.

	Age, years	BMI, kg/m^2^	SBP, mmHg	DBP, mmHg	MAP, mmHg	HR, bpm	HR% of HRmax	RPP, bpm•mmHg	Pedaling power, watts
Δ**% of total leukocytes (CD45** ^**+**^ **)**									
CD3^+^	^−^.2077	^−^.5828	^−^.5786	.0784	^−^.1553	.5707	.4677	.0521	^−^.0361
CD4^+^	^−^.5515	^−^.6390	^−^.8580*	^−^.2919	^−^.4835	.5238	.1931	^−^.3201	.3471
CD8^+^	.0433	^−^.4725	^−^.3525	.2875	.0457	.5177	.5778	.0975	^−^.2635
CD4^+^CD8^+^	.5740	^−^.0363	.6948	.8950**	.8698*	^−^.0696	.3051	.5521	^−^.9103**
CD4^−^CD8^-^	.0292	^−^.3517	^−^.0194	.4170	.2908	.7172	.7566*	.4442	^−^.3140
CD19^+^	^−^.2979	^−^.0133	^−^.8104*	^−^.8947**	^−^.9672***	^−^.1627	^−^.3689	^−^.8118*	.5900
CD56^+^	.1992	^−^.3525	.2276	.8509*	.6794	.4828	.6623	.5876	^−^.5231
CD56^+^CD16^+^	.1921	^−^.2909	.3560	.9196**	.7851*	.4154	.5931	.6547	^−^.5131
CD56^+^CD16^−^	.1627	^−^.3992	^−^.1739	.4148	.1990	.5147	.6479	.2383	^−^.3921
CD64^−^ SSC-A^high^	.7595*	.8014*	.9029**	.4181	.6011	^−^.3277	.1363	.5088	^−^.5666
CD64^+^	.5373	.7241	.8037*	.2490	.4626	^−^.4816	^−^.1625	.3199	^−^.3522
CD14^+^CD16^+^	.6915	.6164	.9404**	.5906	.7598*	^−^.2968	.1288	.5763	^−^.6777
CD14^+^CD16^−^	.4722	.7343	.7312	.1308	.3532	^−^.5164	^−^.2417	.2310	^−^.2455
CD14^−^CD16^+^	.5508	.0827	.6095	.9167**	.8397*	.3042	.6937	.7594*	^−^.6874

**Significant** *p* **-values**; *<.05, **<.01, ***<.001.

**Abbreviations**; SBP, systolic blood pressure; DBP, diastolic blood pressure; HR, heart rate; RPP, rate pressure product; MAP, mean arterial pressure.

The correlations are calculated with exercising values of blood pressure, heart rate, RPP, and MAP.

## Discussion

In the present study, we determined the effect of a short acute cycling exercise bout on blood immune cell proportions in lymphoma patients who had not yet started their cancer treatments. We found that acute exercise had statistically significant effect on CD8^+^ T cells and NK cells. The proportion of total NK cells and CD56^+^CD16^+^ NK cells of total leukocytes increased immediately after exercise and decreased back to baseline at 30 min post-exercise. Further, the proportion of CD8^+^ T cells of total T cells increased immediately after exercise and decreased to baseline at 30 min post-exercise.

The importance of exercise in cancer prevention and control is increasingly recognized. The effect of exercise on lymphoma risk or survival has not been extensively studied, but a suggestive inverse association has been found between leisure-time physical activity and cancer risk in non-Hodgkin lymphomas (Moore et al., 2016). In addition, it has been found that higher physical activity at diagnosis is associated with improved survival in patients with lymphoma (Pophali et al., 2018). Current knowledge suggests that molecular changes that are immediately induced by exercise and repeated over time, are responsible for most of the anti-tumoral benefits of exercise (Wang and Zhou, 2021). Based on preclinical studies, it is hypothesized that exercise-induced mobilization of immune cells plays an important role in the anti-tumor effect (Pedersen et al., 2016; Rundqvist et al., 2020). In this clinical exercise study, we found that cytotoxic T cells and NK cells mobilize significantly in lymphoma patients. The proportion of CD8^+^ T cells of total T cells, and the proportions of total NK cells and CD56^+^CD16^+^ NK cells of total leukocytes increased immediately after acute exercise and decreased back to baseline at 30 min post-exercise, which may indicate that the cells migrate to different tissues after acute mobilization. Krüger et al., (Krüger et al., 2008) studied exercise-induced T cell redistribution in mice and found that bone marrow, lungs, and Peyer’s patches serve as target organs for lymphocytes after exercise. After the exercise, these cells could perhaps also migrate to the tumor site, but this phenomenon cannot be proven based on this study. Previously, however, increased tumor infiltration by both NK cells and cytotoxic T cells in response to exercise has been observed in animals (Pedersen et al., 2016; Rundqvist et al., 2020). Moreover, Bigley et al., found that exercise augments the ability of NK cells to kill HLA-E expressing lymphoma cells during the recovery phase of exercise (Bigley et al., 2014).

CD4^+^ T cell response to acute exercise is generally smaller than that of CD8^+^ cells which results to depressed CD4^+^/CD8^+^ ratios (Shek et al., 1995). In the present study, we found that the proportion of CD4^+^ cells of total T cells decreased significantly immediately after exercise and increased back to baseline at 30 min post-exercise. This is a typical pattern within the lymphocyte pool, as proportions of NK cells and CD8^+^ T cells are known to be increased and CD4^+^ T cells decreased by exercise in healthy individuals (Rowbottom and Green, 2000). In this study, the proportions of CD4^+^CD8^+^ T cells or CD4^−^CD8^−^ T cell did not change. Furthermore, the proportions of CD19^+^ B cells, granulocytes, and monocytes remained unchanged, although in healthy individuals, exercise has been seen to increase the number of these cells to varying degrees (Nielsen et al., 1996; Simpson et al., 2009; Turner et al., 2016). The effect of exercise on immune cells in lymphoma patients has not been studied before, but similar studies have been conducted with other hematologic malignancies. A study with chronic lymphocytic leukemia patients found elevated total lymphocyte count and unchanged CD4^+^ T cell count after 45–60-min exercise (Perry et al., 2012), and another study with pediatric lymphoblastic leukemia survivors found increased neutrophil count after 30-min exercise (Ladha et al., 2006). Acute exercise induces the mobilization of immune cells largely through adrenergic mechanism (Graff et al., 2018), so a smaller density of adrenergic receptors on B cells and CD4^+^ T cells compared to CD8^+^ T cells and NK cells (Murray et al., 1992) may explain their lower mobilization potential observed in this and previous studies. Another explanation for why we did not observe significant changes in cells other than CD8^+^ T cells and NK cells might be the intensity and/or duration of exercise used in the present study. Exercise studies with healthy volunteers usually use moderate to high exercise intensities and fairly long durations (Pistillo et al., 2013; Simpson et al., 2009). In the present study, patients evaluated the exercise intensity either as low or as moderate on Borg scale, and the duration of the exercise was only 10 min, which might have been too short for greater immune cell responses. Previous studies have reported that the effect of exercise is in fact dependent on the intensity, duration, and type of exercise (Da Silva Neves et al., 2015; LaVoy et al., 2017; Schlagheck et al., 2020). Consistent with these findings, we also found that change in CD4^+^CD8^+^ T cell, CD4^−^CD8^−^ T cell, different monocyte subset, and CD56^+^CD16^+^ NK cell proportions correlated positively with surrogates of exercise intensity such as exercising blood pressure and rate pressure product.

Our study has certain limitations. No individual maximal fitness tests were performed, so we were unable to determine the same relative intensity of exercise stress for each patient. Although most patients rated the exercise exertion on the Borg scale as moderate, the exercise load was individually chosen for each patient. This may contribute to the heterogeneity of the study results obtained between patients. One specific methodological limitation is that the flow cytometer Fortessa was set out to draw 150 μL of the sample each time but Fortessa is not volumetric in nature, which might have resulted in imprecisions in absolute cell counts and higher variation between the samples. Therefore, instead of absolute cell counts, the main figures represent cell proportions that are not sensitive to variation in acquired sample volume. Changes in cell counts are, however, presented as Supplementary Material with methodological considerations. Considering these limitations and the lack of a healthy control group, the present results cannot be directly compared with other studies or non-oncology settings. However, the main aim was to examine the exercise-induced immune cell mobilization on an individual level, which appears to be evident with the applied protocols. All samples from each patient were processed and measured simultaneously, which improves the reliability to estimate exercise-induced changes in leukocytes. Finally, we did not include clinical differential blood count or examine the functional activity of immune cells in the present study, which could have provided more insight on the topic.

## Conclusion

Our aim was to investigate whether acute exercise has an effect on immune cell proportions in lymphoma patients. We found that the proportion of cytotoxic T cells of total T cells and proportions of total NK cells and CD56^+^CD16^+^ NK cells of total leukocytes increased significantly immediately after acute exercise and decreased to baseline levels at 30 min post-exercise. This study shows a potentially useful clinical finding that a short bout of exercise is sufficient to mobilize cytotoxic T cells and NK cells in lymphoma patients. In the future, the research should be replicated with a larger study sample to potentially expand these results. Future research is also needed to understand the association between immune cell mobilization and clinical outcome. It is also important to determine the best intensity of exercise for exercise therapy, although it seems based on our study, that fairly short duration and low-to-moderate exercise intensity are enough to induce important changes in immune cell proportions in lymphoma patients.

## Data Availability

The raw data supporting the conclusion of this article will be made available by the authors, without undue reservation.
