# Supplementary material for: Acute exercise mobilizes CD8+ cytotoxic T cells and NK cells in lymphoma patients
Source: Front Physiol. 2023 Jan 11;13:1078512. doi: 10.3389/fphys.2022.1078512 (PMC9873989; doi:10.3389/fphys.2022.1078512)
Supplement: Supplementary file 1 [file DataSheet1.docx]

**Acute exercise mobilizes CD8^+^ cytotoxic T cells and NK cells in lymphoma patients**

Tiia Koivula^1^, Salla Lempiäinen^1^, Petteri Rinne^2^, Maija Hollmén^3^, Carl Johan Sundberg^4,5^, Helene Rundqvist^6^, Heikki Minn^7^, Ilkka Heinonen^1^

^1^Turku PET Centre, University of Turku and Turku University Hospital, Turku, Finland; ^2^Institute of Biomedicine, University of Turku, Turku, Finland; ^3^MediCity Research Laboratory, University of Turku, Turku, Finland; ^4^Department of Physiology and Pharmacology, Karolinska Institutet, Stockholm, Sweden; ^5^Department of Learning, Informatics, Management and Ethics, Karolinska Institutet, Stockholm, Sweden; ^6^Department of Laboratory Medicine, Karolinska Institutet, Stockholm, Sweden; ^7^Department of Oncology and Radiotherapy, Turku University Hospital, Turku, Finland

**Supplementary material**

**Methods and methodological considerations**

Blood samples were analyzed by running 150 µL of each sample with a BD LSR Fortessa™ flow cytometer. Since the original sample size of 100 µL blood was diluted to 300 µL and only 150 µl of each sample was analysed, the number of cells/µl was calculated multiplying the cell count by 2 and dividing by 100. Repeated measurement ANOVA was used to test the changes in immune cell counts. The associations between total leukocyte counts determined with different methods, and between age, BMI and exercise intensity variables and immune cell counts were examined by Pearson´s correlation.

The absolute counts of immune cells are important to report, although we acknowledge that the number of some immune cells in this study is considerably lower than typically found in healthy individuals, which may be due to cancer, or the methods used in this study. One specific methodological limitation is that the flow cytometer Fortessa is not volumetric in nature, which may have resulted in imprecisions in absolute cell counts. However, the likelihood of Fortessa leading to inaccuracies in absolute cell counts is small. Other methods used, such as washing the samples, is likely to have a greater effect on the number of cells. Lastly, acute exercise can alter plasma volume due to sweating and increased muscle volume due to shift in fluids, affecting the concentrations of immune cells in the blood. However, none of the patients had visible sweating due to short duration and fairly low intensity of exercise. If plasma volume had changed during exercise, the change would be reflected in an increase in the numbers of all immune cells studied, and also at the 30-min post-exercise time point, as patients were not drinking any liquids between blood sample withdrawals.

**Results**

There was no change in the number of total leukocytes (CD45^+^) or in CD19^+^ B cells (Fig. 1A, B). The number of total NK cells increased by 83 % (p<0,01), CD56^+^CD16^+^ NK cells by 109 % (p<0,05) and CD56^+^CD16^-^ NK-cells by 39 % (p<0,05) immediately after exercise and all decreased back to baseline levels at 30 minutes post-exercise (Fig. 1C-E). Total number of T cells (CD3^+^) increased relatively little immediately after exercise but decreased significantly 30 minutes post-exercise (p<0,05) (Fig. 2A). The number of CD8^+^ cytotoxic T cells increased by 36 % (p<0,01) immediately after exercise and decreased back to baseline levels at 30 minutes post-exercise (Fig 2B). The number of CD4^+^ T helper cells, or the number of CD4^+^CD8^+^ double positive T cells, or the number of CD4^-^CD8^-^ double negative T cells did not change (Fig.2C-E). Furthermore, CD4^+^/CD8^+^ ratio decreased immediately after exercise (p<0,01) and increased back to baseline at 30 minutes post-exercise (Fig. 2F). There was no change in granulocyte or monocyte counts (Fig. 3A-E).

There was a significant positive correlation (r=0.9045, p=0.0133) between CD45^+^ cell count (measured by flow cytometry) and total leukocyte count measured by a hematology analyzer (Sysmex XN instrument) even if blood samples for clinical total leukocyte count analysed by Sysmex were not taken at the same time point with other blood samples in the study, but within 1-2 weeks of the study visit. Age and exercising systolic blood pressure correlated positively with change in CD64^+^, CD14^+^CD16^+^, and CD14^+^CD16^-^ monocytes (Table 1). BMI correlated negatively with change in CD56^+^ NK cells (Table 1). Exercising diastolic blood pressure correlated positively with change in CD14^-^CD16^+^ monocytes and exercising heart rate correlated negatively with change in CD4^+^CD8^+^ T cells (Table 1). Finally, age correlated negatively with baseline levels of total leukocytes, thus CD45^+^ cells (r= -0,7658, p=0,0447) and CD64^+^, CD14^+^CD16^-^ and CD14^-^CD16^+^ monocytes (r=-0.9164, p=0.0037, r= -0,8534, p= 0,0146, r=-0,8886, p=0,0075, respectively). BMI correlated negatively with baseline levels of CD14^+^CD16^+^ and CD14^-^CD16^+^ monocytes (r=-0,7861, p=0,0361, r=-0,8246, p=0,0225, respectively).

**
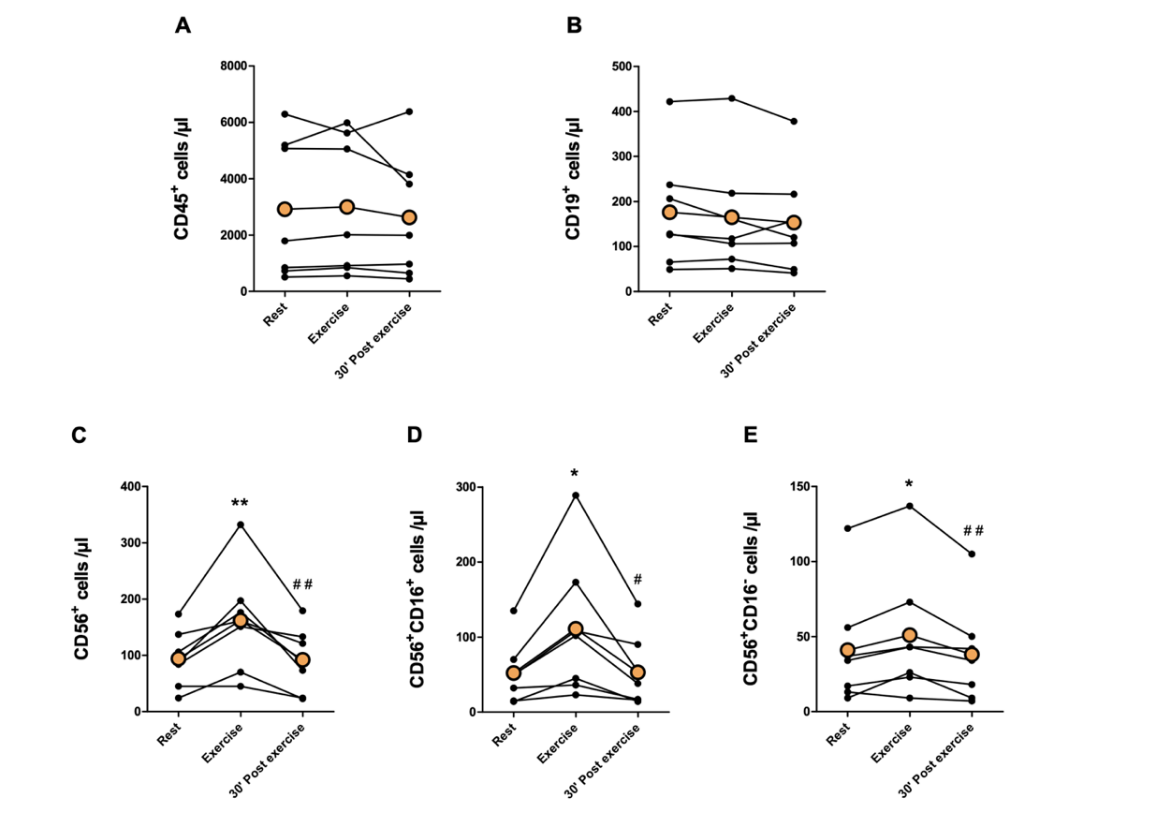
**

**Figure 1.** Changes in (A) total leukocyte, (B) B cell, and (C-E) NK cell levels with acute exercise. Orange points represent the mean. *p<0,05 between rest and exercise; **p<0,01 between rest and exercise; #p<0,05 between exercise and 30 minutes post-exercise; ##p<0,01 between exercise and 30 minutes post-exercise.

**
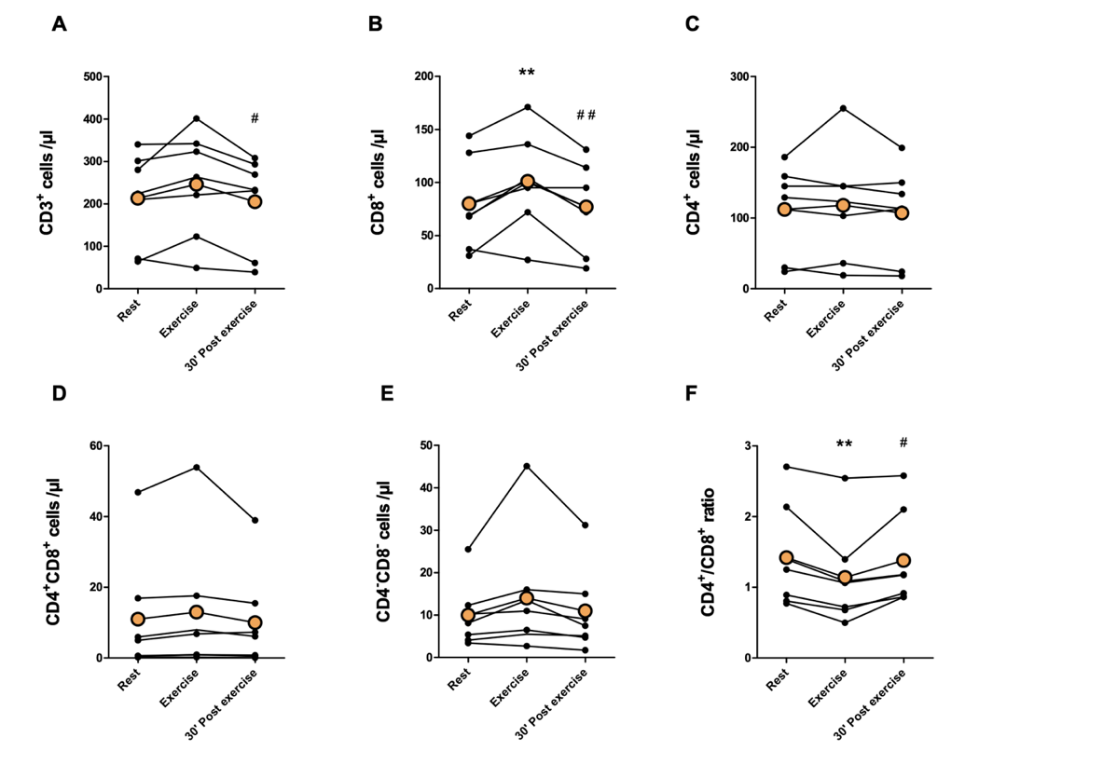
**

**Figure 2.** Changes in (A) total T cell, (B) CD8^+^ T cells, (C) CD4^+^ T cell, (D) CD4^+^CD8^+^ T cell, and CD4^-^CD8^-^ T cell levels, and in (F) CD4^+^CD8^+^ ratio with acute exercise. Orange points represent the mean. **p<0,01 between rest and exercise; #p<0,05 between exercise and 30 minutes post-exercise; ##p<0,01 between exercise and 30 minutes post-exercise

**
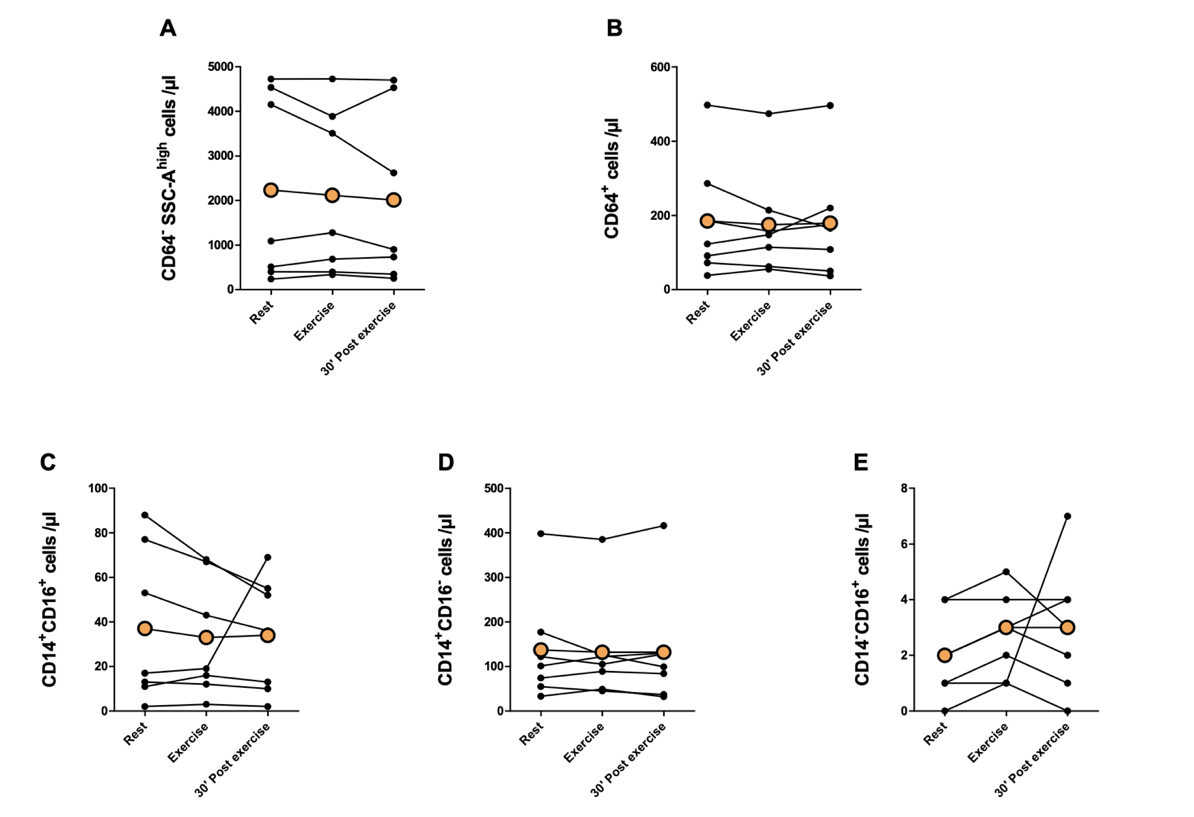
**

**Figure 3.** Changes in (A) granulocyte levels and in (B) total monocyte, (C) CD14^+^CD16^+^ monocyte, (D) CD14^+^CD16^-^ monocyte, and (E) CD14^-^CD16^+^ monocyte levels with acute exercise. Orange points represent the mean.

| **Table 1. Pearson correlation between age, BMI, and exercise intensity variables and change in the cell number between rest and exercise.** | | | | | | | | |  | |  | |  | |  | |  | |  | | |  | |  | |  | |
| --- | --- | --- | --- | --- | --- | --- | --- | --- | --- | --- | --- | --- | --- | --- | --- | --- | --- | --- | --- | --- | --- | --- | --- | --- | --- | --- | --- |
|  | Age, years | BMI, kg/m^2^ | SBP, mmHg | DBP, mmHg | MAP,  mmHg | HR,  bpm | HR% of HRmax | | | | | | RPP,  bpm•mmHg | | | | | | | Pedaling power, watts | | | | | | |  |
| ΔCD45^+^ | -0.3433 | -0.0644 | 0.0310 | -0.1646 | -0.0348 | 0.4284 | 0.1897 | | | | | | 0.2763 | | | | | | | 0.3352 | | | | | | |  |
| ΔCD3^+^ | -0.6179 | -0.4811 | -0.6008 | -0.2489 | -0.2842 | 0.6972 | 0.3034 | | | | | | -0.0019 | | | | | | | 0.3507 | | | | | | |  |
| ΔCD4^+^ | -0.6494 | -0.2610 | -0.4801 | -0.3584 | -0.2937 | 0.7011 | 0.2763 | | | | | | 0.0876 | | | | | | | 0.4924 | | | | | | |  |
| ΔCD8^+^ | -0.3945 | -0.7052 | -0.6743 | 0.0208 | -0.2015 | 0.5114 | 0.2893 | | | | | | -0.1603 | | | | | | | 0.0583 | | | | | | |  |
| ΔCD4^+^CD8^+^ | 0.3941 | -0.0819 | 0.2721 | 0.0506 | 0.0520 | **-0.7606*** | -0.5493 | | | | | | -0.3580 | | | | | | | -0.5076 | | | | | | |  |
| ΔCD4^-^CD8^-^ | -0.5763 | -0.2806 | -0.2913 | -0.1796 | -0.0954 | 0.6941 | 0.3148 | | | | | | 0.2361 | | | | | | | 0.3485 | | | | | | |  |
| ΔCD19^+^ | 0.2656 | 0.5688 | 0.0427 | -0.3304 | -0.1854 | 0.3392 | 0.4632 | | | | | | 0.2176 | | | | | | | -0.0253 | | | | | | |  |
| ΔCD56^+^ | -0.6446 | **-0.8301*** | -0.4800 | 0.1518 | -0.0372 | 0.1414 | -0.2170 | | | | | | -0.2299 | | | | | | | 0.2885 | | | | | | |  |
| ΔCD56^+^CD16^+^ | -0.6177 | -0.7506 | -0.4421 | 0.1473 | -0.0304 | 0.1017 | -0.2376 | | | | | | -0.2228 | | | | | | | 0.3118 | | | | | | |  |
| ΔCD56^+^CD16^-^ | -0.1335 | -0.5266 | -0.2600 | 0.0561 | -0.0422 | 0.3312 | 0.2361 | | | | | | -0.0090 | | | | | | | -0.1752 | | | | | | |  |
| ΔCD64^-^ | 0.5297 | 0.5641 | 0.6540 | 0.1914 | 0.3730 | 0.0767 | 0.3759 | | | | | | 0.5361 | | | | | | | -0.3847 | | | | | | |  |
| ΔCD64^+^ | **0.8158*** | 0.7408 | **0.7894*** | 0.2722 | 0.4545 | -0.2321 | 0.2429 | | | | | | 0.4443 | | | | | | | -0.6639 | | | | | | |  |
| ΔCD14^+^CD16^+^ | **0.8756**** | 0.7095 | **0.7841*** | 0.3878 | 0.5223 | -0.1158 | 0.4088 | | | | | | 0.5309 | | | | | | | -0.7331 | | | | | | |  |
| ΔCD14^+^CD16^-^ | **0.7653*** | 0.7503 | **0.7638*** | 0.1944 | 0.3975 | -0.2767 | 0.1605 | | | | | | 0.3871 | | | | | | | -0.6089 | | | | | | |  |
| ΔCD14^-^CD16^+^ | 0.2292 | -0.2671 | 0.3201 | **0.8908**** | 0.7458 | 0.2970 | 0.4975 | | | | | | 0.5504 | | | | | | | -0.5522 | | | | | | |  |
| **Significant p-values**; *<0.05, **<0.01  **Abbreviations**; SBP = systolic blood pressure; DBP = diastolic blood pressure; HR = heart rate;  RPP = rate pressure product; MAP = mean arterial pressure  The correlations are calculated with exercising values of blood pressure, heart rate, RPP, and MAP. | | | | | | | |  | |  | |  | |  | |  | |  | | |  | |  | |  | |  |
